# Radioiodine Contamination Artifacts and Unusual Patterns of Accumulation in Whole-body I-131 Imaging: A Case Series

**DOI:** 10.5812/ijem.9329

**Published:** 2014-01-01

**Authors:** Pelin Ozcan Kara, Emel Ceylan Gunay, Alihan Erdogan

**Affiliations:** 1 Department of Nuclear Medicine, Faculty of Medicine, Mersin University , Mersin, Turkey

**Keywords:** Thyroid Cancer, Radioiodine Scan, False Positive

## Abstract

**Introduction:**

Radioactive iodine has been used for more than 50 years for the treatment of thyroid diseases. Differentiated thyroid cancers have the ability to trap iodine. Therefore, radioiodine can be used both diagnostically and therapeutically. In the follow-up of patients, it is critical to interpret radioiodine scans correctly.

**Case Presentation:**

Non-physiological Iodine-131 (I-131) extra-thyroidal uptake detected on post-therapy or diagnostic I-131 scanning are not always interpreted as functioning metastatic thyroid cancer.

**Conclusions:**

This study provides detailed information and case samples of radiodine contamination artifacts and non-physiological, non-metastatic extra-thyroidal I-131 accumulation in whole-body I-131 imaging.

## 1. Introduction

Thyroid cancers are classified as differentiated and undifferentiated according to their histological appearance. Differentiated thyroid carcinoma (DTC) is an uncommon disease, defined as a carcinoma deriving from follicular epithelium. Papillary cancer comprises about 85 % of cases. The prognosis of disease is generally excellent with appropriate treatment. Total thyroidectomy, followed by radioiodine therapy and thyroid-stimulating hormone (TSH) suppressive thyroid hormone therapy, is the standard treatment of differentiated thyroid cancer. Radioiodine ablation is a post-surgical adjuvant modality. Radioiodine therapy is indicated to destroy microscopic cells remaining after surgery as well as to increase the specificity of serum thyroglobulin (Tg). Thyroid cancers have the ability to trap iodine and produce Tg. TSH-stimulated Tg should be negative (< 0.5 ng/mL) after surgery and radioiodine therapy (1). Ablation also allows sensitive “post-therapy” whole-body scintigraphy (Rx WBS) that may detect previously occult metastases. However, the accurate evaluation of radioiodine scans is critical in the management of patients with thyroid cancer. Anatomical and physiological variants as well as artifacts and non-physiological accumulation of I-131 mimicking metastatic disease must be recognized to avoid incorrect management.

## 2. Cases Presentation 

One of the causes of false positivity in I-131 whole-body scans is contamination with body secretions and awareness of the possibility of contamination would be obligatory in reporting whole-body I-131 scans.

Radioiodine accumulation because of local contamination reported in the literature includes sites such as hair, skin, and clothes. The aim of this study was to provide detailed information and case samples of radioiodine contamination artifacts and non-physiological, non-metastatic extra-thyroidal I-131 accumulation in whole-body I-131 imaging.

### 2.1. Methods

Thyroxin therapy was stopped for four weeks to obtain a serum TSH level above 30 µ-IU/mL. As a matter of routine, we advised patients to adhere to a low iodine diet for ten days prior to radioiodine scanning. The low iodine diet was advised to be continued 24 hours after the radioiodine administration too. We documented negative serum pregnancy test before administration of radioiodine in all women of child bearing age. Diagnostic whole-body scans were performed 48 hours after the administration of 185 MBq I-131 and 2 to 10 days after the administration of therapeutic dose of radioiodine. Radioiodine scans were performed with a dual-head gamma camera equipped with high energy collimators (Siemens, E-cam, IL, USA). Both anterior and posterior whole-body scans and spot views of neck and chest were performed, as routine. Delayed scans were obtained if necessary.

### 2.2. Case 1

**Figure 1.**
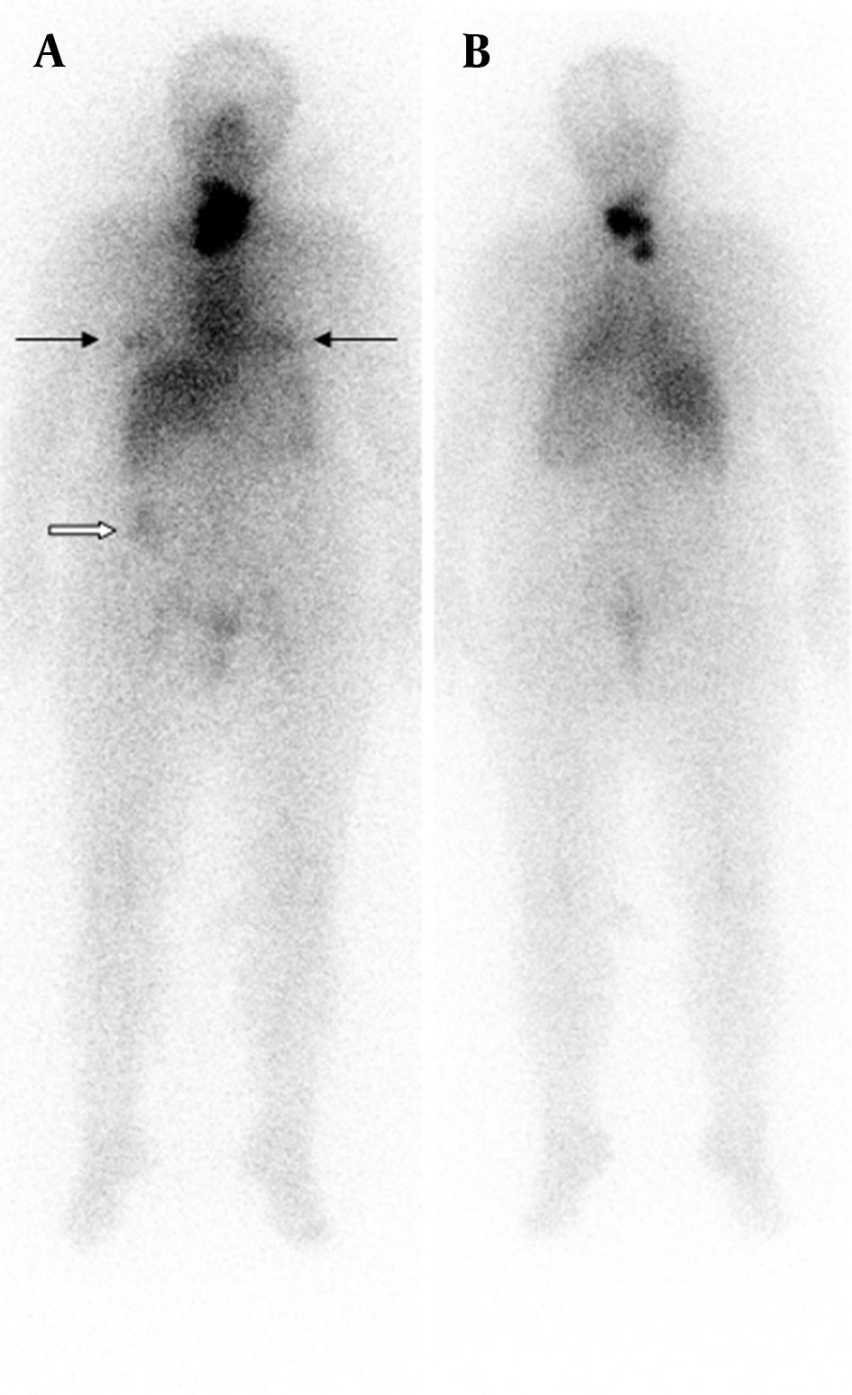
Anterior and Posterior Whole-body Scan Images 5 Days after Therapy Demonstrating Intense Accumulation of Radioiodine at Thyroid Bed with Physiological Bilateral Breast and Colon Activity Teaching point: Physiological bilateral breast and colon activity may be seen in radioiodine scan.

**Figure 2.**
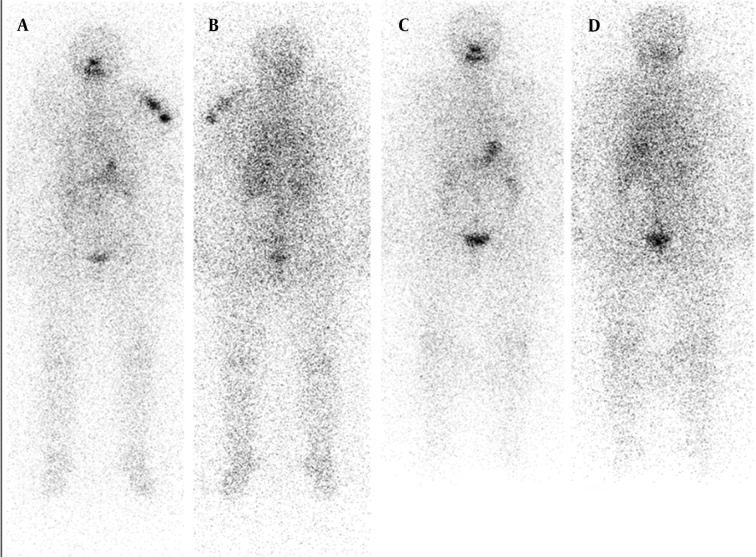
Anterior and Posterior Whole-body Scan Images on Follow-up Scan After Two Years of I-131 Therapy A and B show intense accumulation of I-131 in left humerus mimicking metastatic disease. There was no uptake in thyroid bed demonstrating successful ablation of the remnant. C, Anterior and D, Posterior show that whole-body scan images were normal after having a bath and removing head scarf. There was physiological activity in the bowel and stomach in both scans.

A 57-year-old women underwent total thyroidectomy and radioiodine ablation therapy two years ago. Pathology report demonstrated widely invasive follicular thyroid carcinoma. Whole-body scan findings at day 5 after the administration of 100 mCi radioiodine therapy demonstrated intense accumulation of radioiodine in thyroid bed. Additionally, bilateral physiological breast iodine uptake (black arrows in Figure 1 A) and colon activity (white arrow in Figure 1 A) were seen. A diagnostic scan was performed after withholding thyroxin therapy for four weeks on follow-up two years after the therapy. Her serum TSH and Tg levels were 115 µ-IU/mL and > 0.2 ng/mL, respectively when the diagnostic scan was acquired. The diagnostic whole-body scan was performed 48 hours after administration of 185 MBq I-131 and showed accumulation of radioiodine in her left humerus mimicking metastatic disease (Figure 2 A and 2 B). There was no evidence of metastatic disease after 72 hours (Figure 2 C and 2 D).

### 2.3. Case 2

**Figure 3.**
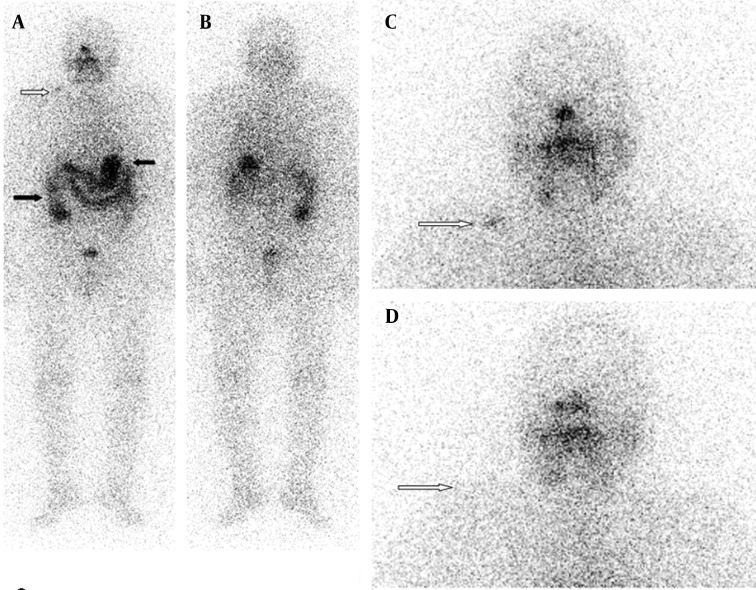
Anterior and Posterior Whole-body Scan Images on Follow-up Scan Eleven Years After the Diagnosis

The patient was a 74-year-old man on follow-up with papillary thyroid cancer for 11 years. A diagnostic radioiodine scan was performed after withholding thyroxin therapy for four weeks. His serum TSH and Tg levels were 100 µ-IU/mL and < 0.1 ng/mL, respectively when the diagnostic scan was acquired. The diagnostic whole-body scan was acquired 48 hours after administration of 185 MBq I-131 and showed accumulation of radioiodine in his right shoulder mimicking metastatic disease (white arrows in Figures 3A and 3C) and intense bowel activity (black arrows in Figures 3A and 3B). There was no evidence of metastatic disease after self-cleaning after 72 hours (white arrow in Figure 3 D). Although radioiodine uptake due to contamination is usually outside the body contours, superposition with body structures may lead to misinterpretation. Radioiodine scans should be interpreted carefully. Delayed images after self-cleaning and changing clothes are extremely useful.

### 2.4. Case 3

**Figure 4.**
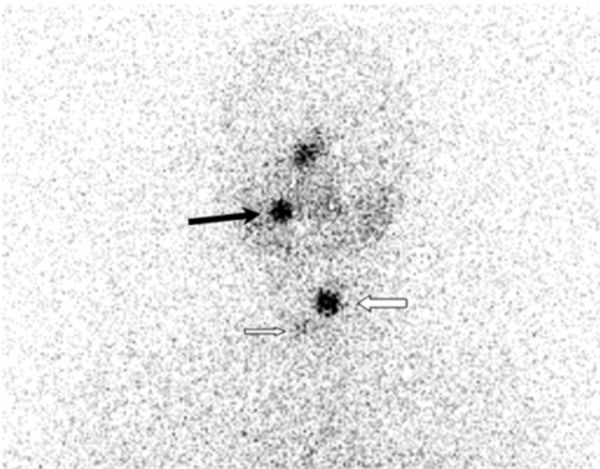
Anterior Neck Spot View Uptake in thyroid bed is demonstrated with white arrows. Additionally, focal accumulation of radioiodine is seen in right maxillary region (black arrow) that belonged to a dental inflamation. See physiological nasal activity.

A 48-year-old woman was referred to our clinic for follow-up of her papillary thyroid cancer. Diagnostic radioiodine scan was performed after withholding thyroxin therapy with a serum TSH level of > 150 µ-IU/mL and serum Tg level of < 0.2 ng/mL and demonstrated a focal accumulation of radioiodine in her right maxillary region belonged to a dental inflamation and physiological nasal radioiodine activity (Figure 4). 

### 2.5. Case 4

**Figure 5.**
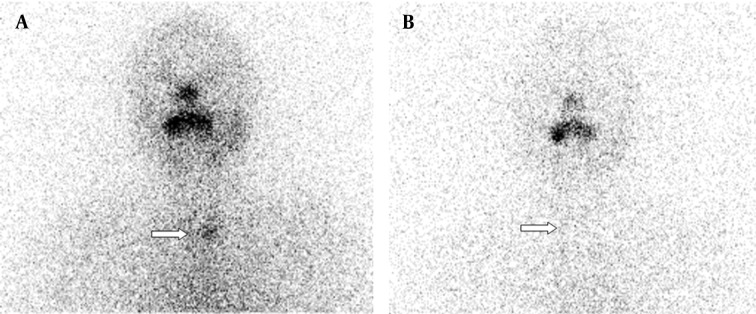
Anterior Neck Spot View A, Uptake inferior to left thyroid bed that belonged to oesophageal activity is shown with white arrows. B, Uptake is cleaned with a drink of water.

A 66-year-old woman was referred to our clinic for follow-up of her papillary thyroid cancer. Diagnostic radioiodine scan was performed after withholding thyroxin therapy with a serum TSH level of > 30 µ-IU/mL and serum Tg level of < 0.2 ng/mL and demonstrated a focal accumulation of radioiodine belonged to oesophageal activity (Figure 5A). After drinking water there was not any activity (Figure 5B). 

Teaching point: Although oesophageal activity usually appears as a linear midline region of increased I-131 uptake that can be reduced by drinking fluids, focal radioiodine accumulation in neck should be kept in mind to be a physiological oesophageal activity. Contamination artifacts may always occur as a result of radioactive sweat, saliva, or urine. Contaminations can easily be prevented by taking simple precautions.

### 2.6. Case 5

The patient was a 64-year-old woman whom was followed-up because of papillary thyroid cancer and endometrial carcinoma. She had undergone lympadenectomy. After withholding thyroxin therapy, her serum TSH and Tg levels were 75 µIU/mL < 0.2 ng/mL, respectively. There was no pathological radioiodine uptake in whole-body scan images except for physiological uptake in bowel and stomach. There was diffuse uptake in left lump because of lymphedema.

### 2.7. Case 6

A 15-year-old girl was referred to our clinic for ablation therapy of follicular thyroid carcinoma after total thyroidectomy. While her serum TSH and Tg levels were 229 µIU/mL and 0.2 ng/mL, respectively after radioiodine therapy, radioiodine scan was performed. There was a focal accumulation of radioiodine in thyroid bed. Additionally, less intense accumulations of radioiodine were detected in left maxillary and upper mediastinal regions.

## 3. Conclusions

**Figure 6.**
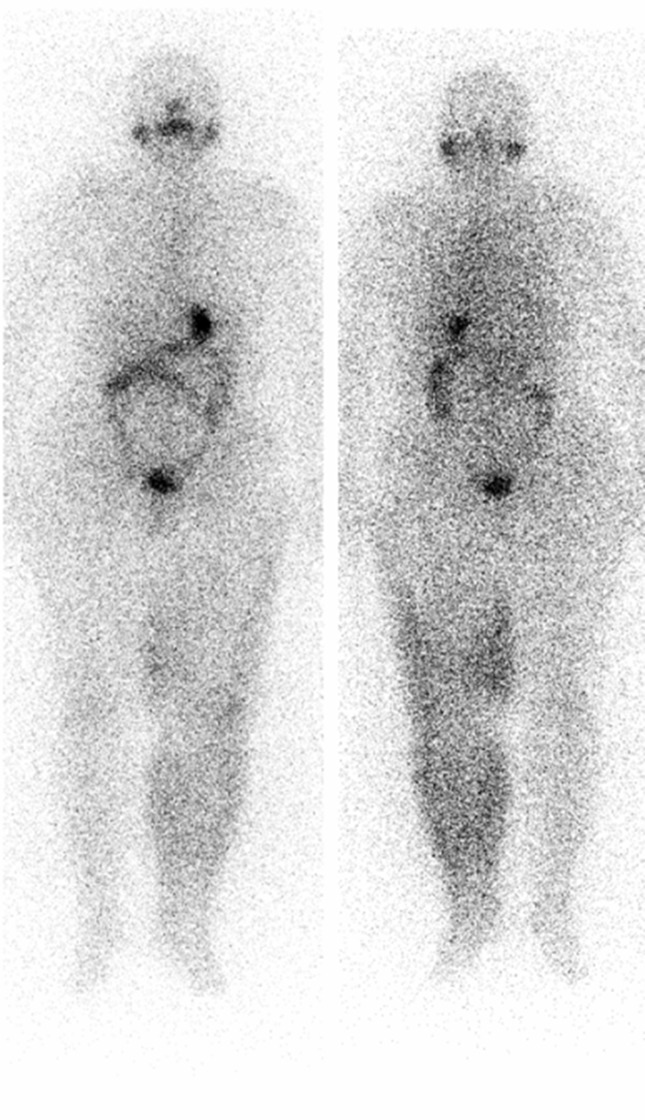
Anterior and Posterior Whole-body Scan Images Demonstrating No Pathological Radioiodine Uptake. Diffuse uptake in left lump belongs to lymphedema.

**Figure 7.**
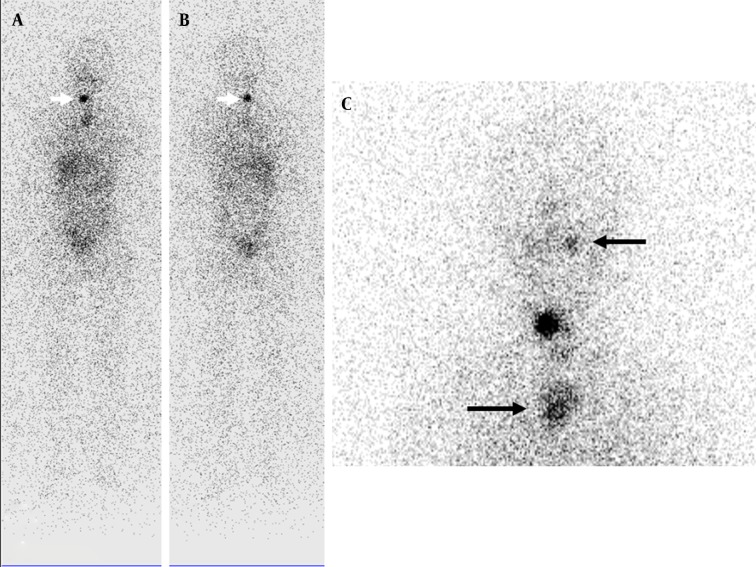
Anterior and Posterior Whole-body Scan Images and Neck Static Image Demonstrating Radioiodine Accumulation in Thyroid Bed . Less intense activities in left maxillary and upper thoracic regions were seen that belonged to dental inflamation and thymic activity, respectively (black arrows). Thoracic computed tomography scan was performed after the radioiodine scan showed a heterogeneous mass in the mediastinum, suggestive of thymus.

The accurate interpretation of radioiodine scans is critical in the management of patients with thyroid cancer. Anatomic and physiological variants as well as artifacts and non-physiological accumulation of I-131 mimicking metastatic disease must be recognized to avoid incorrect management. Previously, a variety of cases illustrating true positive, true negative, and false positive findings were presented in a review article (2). Although false positive findings do occur, the specificity of radioiodine scans is greater than 90% (2). False positive scans result from physiological (Figures 1 and 5) and non-physiological uptake due to a benign or pathologic process (Figures 4 and 6), and also contaminations (Figures 2 and 3). Uptake in thyroid bed is usually seen because of the remnant tissue after total thyroidectomy (Figure 4). When evaluating the scan, ectopic thyroid tissue uptake should also be kept in mind. The urinary bladder is normally seen in whole-body scan because of the excretion of most of the radioiodine through the kidneys. Salivary, mammary, and sweat glands as well as gastric mucosa, choroid plexus, and placenta are known to concentrate iodine (3). Therefore, radioiodine is found not only in sweat, saliva, nasal and gastric secretions, and cerebrospinal fluid, but also in breast milk and foetal blood (4). Radioiodine uptake in the salivary, mammary (Figure 1), and sweat glands as well as gastrointestinal (Figures 1-3, 6) and urinary glands should be decided as physiologic while contamination must be considered outside these regions. The most important potential source of error in Rx WBS is local contamination (clothing, skin, hair, collimator, crystal, etc.) followed by esophageal activity (Figure 5), asymmetrical salivary gland uptake, non-specific uptakes e.g. pulmonary infections, edema (Figure 6), breasts (Figure 1), kidney cysts, and the thymus (Figure 7). Contaminations are superficial and usually cleaned by washing the contaminated area or changing clothes. Superposition with body structures may lead to misinterpretation (Figure 2). Lateral images may be helpful as well as self-cleaning of the patient.

Contamination of hair when styling with saliva, contamination of the neck due to drooling during sleep, and contaminated chewing gum have been shown as false positive sites of I-131 localisation (5). A previous study reported two cases involving the sequestration I-131 contaminated handkerchiefs in patients’ pockets (6). To avoid artifacts caused by cutaneous contamination with radioiodine, the patient should shower and change underwear before Rx WBS. The contamination of contact lenses during radioiodine therapy was also reported by another study (7). The maximum I-131 secretion was within the first day and it decreased markedly in the following days. The radiation dose caused to the accumulation of radioiodine in the patient’s contact lenses was found to be negligible in comparison to the dose in the surrounding tissue. One of the complications of radioiodine therapy is sialadenitis. In patients who have I-131 uptake around the major salivary glands radiation sialadenitis should be considered. The authors reported 2 examples of unusual false-positive I-131 whole-body scan findings in papillary thyroid carcinoma caused by chronic sialadenitis of a submandibular gland and a maxillary mucous retention cyst (8). Increased superﬁcial I-131 uptake in the left calf suggesting contamination was reported to be a dilated left greater saphenous vein by a prior study in an interesting image report (9). Mediastinal radioiodine uptake due to hiatal hernia was also reported by another study as one of the false positive findings in radioiodine scans (10). Increased radioiodine uptake was reported in the lacrimal sac in a patient with dacryocystitis and in the orbit of a patient with a glass eye (11). Recently, we presented an interesting contamination artifact image in a patient with intense I-131 accumulation in the anterolateral aspects of her neck and breasts that was due to a bra contamination and manifested on I-131 images (12). In another case report with review of the literature, technetium-99m pertechnetate, technetium-99m MDP methylene diphosphonate, and radioiodine imaging were featured in a 33-year-old male patient with metastatic insular carcinoma of the thyroid (11). Widespread contamination mimicking distant metastases was presented in a patient with papillary microcarcinoma in whole-body images obtained after iodine-131 ablation therapy (12).

### 3.1. Perspective

The accurate interpretation of radioiodine scans is critical in the management of patients with thyroid cancer. When evaluating radioiodine scans, delayed images after self-cleaning and changing clothes, and patient’s clear clinical history are extremely useful. Contamination artifacts may always occur as a result of radioactive sweat, saliva, or urine. Contaminations can easily be prevented by taking simple percautions. There is also need for further studies demonstrating the value of single-photon emission computed tomography and computed tomography imaging especially for contamination artifacts.
